# Regulatory T cells in multiple sclerosis and myasthenia gravis

**DOI:** 10.1186/s12974-017-0892-8

**Published:** 2017-06-09

**Authors:** K. M. Danikowski, S. Jayaraman, B. S. Prabhakar

**Affiliations:** Department of Microbiology and Immunology, University of Illinois at Chicago, Chicago, IL 60612 USA

**Keywords:** Regulatory T cell, Treg, Myasthenia gravis, Multiple sclerosis, Autoimmune disease, FoxP3, Migration, Dysfunction, Suppression

## Abstract

Multiple sclerosis (MS) is a chronic debilitating disease of the central nervous system primarily mediated by T lymphocytes with specificity to neuronal antigens in genetically susceptible individuals. On the other hand, myasthenia gravis (MG) primarily involves destruction of the neuromuscular junction by antibodies specific to the acetylcholine receptor. Both autoimmune diseases are thought to result from loss of self-tolerance, which allows for the development and function of autoreactive lymphocytes. Although the mechanisms underlying compromised self-tolerance in these and other autoimmune diseases have not been fully elucidated, one possibility is numerical, functional, and/or migratory deficits in T regulatory cells (Tregs). Tregs are thought to play a critical role in the maintenance of peripheral immune tolerance. It is believed that Tregs function by suppressing the effector CD4+ T cell subsets that mediate autoimmune responses. Dysregulation of suppressive and migratory markers on Tregs have been linked to the pathogenesis of both MS and MG. For example, genetic abnormalities have been found in Treg suppressive markers CTLA-4 and CD25, while others have shown a decreased expression of FoxP3 and IL-10. Furthermore, elevated levels of pro-inflammatory cytokines such as IL-6, IL-17, and IFN-γ secreted by T effectors have been noted in MS and MG patients. This review provides several strategies of treatment which have been shown to be effective or are proposed as potential therapies to restore the function of various Treg subsets including Tr1, iTr35, nTregs, and iTregs. Strategies focusing on enhancing the Treg function find importance in cytokines TGF-β, IDO, interleukins 10, 27, and 35, and ligands Jagged-1 and OX40L. Likewise, strategies which affect Treg migration involve chemokines CCL17 and CXCL11. In pre-clinical animal models of experimental autoimmune encephalomyelitis (EAE) and experimental autoimmune myasthenia gravis (EAMG), several strategies have been shown to ameliorate the disease and thus appear promising for treating patients with MS or MG.

## Background

Multiple sclerosis (MS) and myasthenia gravis (MG) are autoimmune diseases affecting the central nervous system (CNS) and the neuromuscular junction (NMJ), respectively. These diseases are characterized by inflammation, immune dysregulation, and immune over activity [1, 2]. Defects in self-tolerance leading to autoimmunity distinguish MS from other well-known neuroinflammatory diseases including Parkinson’s disease, Alzheimer’s disease, and stroke episodes [3]. Likewise, the autoimmune component of MG distinguishes it from many other muscular dystrophies [2]. MS and MG affect 50/100,000 and 20/100,000 people, respectively, in the USA; this prevalence amounts to more than the collective prevalence of other neurologically relevant autoimmune diseases including optic neuritis, Guillain-Barre Syndrome (GBS), and acute disseminated encephalomyelitis [4–8]. Current treatments for MS and MG involve non-specific mechanisms of action, do not produce lifelong protection from flare-ups, and have undesirable side effects including fatalities [9–12]. Since T regulatory cells (Tregs) characterized as CD4^+^CD25^+^FoxP3^+^ (forkhead box P3) cells have emerged pivotal in suppressing autoimmune diseases like type 1 diabetes and others, this review is focused on evaluating the role of Treg defects or dysfunctions in MS- and MG-related autoimmune pathology [1, 13–17]. It also discusses the beneficial effects of Treg augmentation as a potential treatment strategy. It does so by summarizing recent data from MS and MG patients, as well as their pre-clinical models: experimental autoimmune encephalomyelitis (EAE) and experimental autoimmune myasthenia gravis (EAMG), respectively.

Although MS and MG are distinct autoimmune diseases, some studies suggest a co-morbidity [18]. A retrospective analysis indicated that five out of 1718 patients with MS also had MG (0.29%; expected percentage (0.005–0.015%)) [18]. Notably, the severity of both MS and MG was mild and the MG symptoms were ocular with none of these patients having positive anti-acetylcholine receptor (AChR) antibodies [18]. In another study, MS preceded MG in five out of eight patients by 6 to 8 years [19]. Onset of MG in MS patients treated with interferon (IFN)-β and glatiramer acetate (GA) was reported in one [20], but not in another study [18]. Therefore, definitive evidence of co-morbidity between MS and MG requires further validation in large independent cohorts of patients.

Despite differences in pathologies of MS and MG, there are immunological similarities. Both MS and MG are considered largely T cell mediated [1, 21]. MS and MG patients have increased numbers of Th1 and Th17 cells along with their associated cytokines IL-1, IL-6, IL-17, IFN-γ, and tumor necrosis factor (TNF)-α (Table 1) [21–26]. Further, Tregs of these patients have numerous documented dysfunctionalities [1, 13, 27, 28]. T cells originate from the thymus, and evidence suggests the thymus as a key regulator in the pathogenesis MG, and less so in MS [18, 20, 29]. Thus, thymectomy is a treatment option from which few MS patients may benefit [30, 31]. Both MS and MG have altered recent thymic emigrants suggestive of lower thymic output (Table 1) [28, 29, 32]. Further, MG and MS thymuses have been found to contain clonally expanded B cell lines (Table 1), a characteristic not found in control patients, or patients with systemic lupus erythematosus (SLE), anther autoimmune disease [33]. Germinal centers appear in the pathogenesis of both diseases [34]. CXCR5, a receptor associated with germinal center (GC) migration, can be found expressed on both T and B cells, but is elevated on CD4^+^ cells in both diseases (Table 1) which correlates well with the disease severity [34]. Both MS and MG patients have evidence of antigen-specific T cells [35, 36]. While MG patients have definitive evidence of antigen-specific antibody-mediated damage to the NMJ, the presence of antigen-specific antibodies in MS is debatable [37–39]. Immunological similarities and differences become more evident with treatments using immune modulating agents. Rituximab, a B cell depleting anti-CD20 antibody, has proven beneficial in both diseases, indicating B cell importance for each [40, 41]. In contrast, MS and MG patients differ in their response to IFN-1 (i.e., alpha and beta) treatments (Table 1). Development of MG after IFN-α treatment has been previously reported, while treatment of MS with IFN-β has been shown to be beneficial [12, 42]. IFN-1 (IFN-β) has also led to increased autoantibody production, a possible reason for MG exacerbation upon IFN-1 (IFN-α) treatment [43]. Similarly, a patient with MS treated with GA was seen to develop MG [44]. Lastly, anti-TNF-α therapy has resulted in different effects in patients with MS and MG. In MS, anti-TNF-α therapy has been unsuccessful despite success in pre-clinical models [45]. Whereas a majority of MG patients receive benefit, although some receive no benefit or even disease exacerbation [45, 46]. These studies clearly indicate that the immunological perturbation in these two diseases may have some common features, but are dissimilar.

**Table 1:** Immunological Comparisons between multiple sclerosis and myasthenia gravis

	Multiple sclerosis	Myasthenia gravis
Similarities	↑ Th1 and Th17 cells [24] ↑ IL-1, -6, -17, IFN-γ, and TNF-α [26] Treg-related genetic polymorphisms (IL-2 signaling, CD25, CD127) [55] ↓ Recent thymic emigrants in blood [29, 32] ↑ CXCR5 expression, correlates with disease [34] Clonally expanded B cells in thymus [33] ↓ Tr1 and IL-10 [27] ↑ Fas expression on Tregs [104]	↑ Th1 and Th17 cells [23] ↑ IL-1, -6, -17, IFN-γ, and TNF-α [23, 25] Treg-related genetic polymorphisms [56] ↓ Recent thymic emigrants in blood [28] ↑ CXCR5 expression, correlates with disease [34] Clonally expanded B cells in thymus [33] ↓ Expression of FoxP3 and IL-10 on Tregs [28] ↑ Fas expression on Tregs [13]
Differences	Thymectomy not beneficial [31] Macrophage cell-mediated damage [96] Can be treated via IFN-1 (beta) [12]	Thymectomy Beneficial [197] Antibody-mediated damage [39] Can be induced via IFN-1 (alpha) [42]

*IFN* interferon, *TNF* tumor necrosis factor, *Th* T helper cell, *Tr1* T-regulatory 1 cell, *Treg* T-regulatory cell

Autoimmune development may not only be influenced by inadequate Treg numbers or defective Treg function, but it is also influenced by effector T cells (Teff; CD4^+^FoxP3^−^) resistant to suppression [47]. Although this review focuses on restoring Treg numbers and deficits, Teff resistance should be briefly discussed. The local cytokine milieu of IL-2, IL-4, IL-6, IL-15, and TNF-α have all been shown to influence Teff resistance to suppression [48, 49]. In MS, a decrease in the frequency of Tregs and resistance of Teffs to suppression were noted [50–52]. Similarly, both Tregs and Teffs from MG patients were found to be defective in ex vivo studies [53]. Whereas FoxP3 inhibited Th17 differentiation via repression of transcription factor RORγt, exogenous provision of IL-6 supported the differentiation of Th17 cells, suggesting the plasticity of the T cell under appropriate conditions [54]. Genetic studies unraveled polymorphisms associated with molecules related to Treg function in MS and MG patients [55, 56]. Although these data suggest an intrinsic functional defect in Tregs (Table 1), it is not clear whether it is sufficient to impair the functionality of Tregs. However, the conversion of FoxP3^+^ Tregs derived from normal humans into Th17 cells under the influence of IL-1 and IL-2 ex vivo has been documented, supporting the plasticity of Tregs [57], also observed in mice [54]. This is also suggested from an experiment in EAMG noting that the Treg defects appear after disease induction but the disease itself can be suppressed upon adoptive transfer of ex vivo generated Tregs [58, 59]. Inasmuch as the Tregs appear to be defective in both MS and MG (Table 1), we have focused this review on both intrinsic and extrinsic factors affecting Treg function in these diseases [1, 13, 27, 28].

## Main text

### Implications of dysregulated Tregs in MS and MG

Tregs play a key role in maintaining self-tolerance, and their dysfunction is well documented in multiple autoimmune diseases including Type 1 diabetes, GBS, psoriasis, and others [1, 13–17]. Tregs regulate immune response in the periphery predominantly by suppressing Teff cells. Although significant differences in the number of circulating Tregs in MS or MG patients relative to healthy controls are not frequently reported, Tregs from these patients are reported to have lower suppressive capabilities [1, 13, 60, 61]. This suggests that functional deficits in Tregs may contribute to the pathogenesis of MS and MG. For example, defects in Treg suppressor molecules have been linked to MS, such as reduced IL-10 production and genetic variations in CD25 [27, 55]. Likewise, MG patients have documented dysregulation in cytotoxic T-lymphocyte-associated protein 4 (CTLA-4) expression, IL-2 sensitivity, and the levels of transforming growth factor beta (TGF-β) gene expression [25, 62, 63]. Mechanistically, lower Treg suppressive capabilities may lead to enhanced production of pro-inflammatory cytokines such as IL-6, IL-17, and IFN-γ, as well as activation of autoantibody producing B cells. Normally, Tregs suppress the production of these pro-inflammatory cytokines through contact-dependent and contact-independent suppression of Teff cells known to produce IL-6, IL-17, and IFN-γ [64, 65]. Functional Tregs are also believed to directly eliminate both antigen-presenting and autoantibody-producing B cells in a contact-dependent manner through secretion of perforin and granzyme as seen in lupus prone mice (NZB/W) [66]. This mechanism may be pivotal for Treg-based amelioration of MG and perhaps MS [2, 66, 67]. The autoreactive B cells can produce autoantibodies against acetylcholine receptor (AChR) or muscarinic receptor, thus causing membrane damage via activation of the complement cascade in MG; likewise, it has been suggested that autoantibodies may also be involved in the pathogenesis of MS [68, 69]. Furthermore, activated B cells are found to be upregulated in the neuroinflammatory sites of the CNS in MS patients and in the blood of MG patients [70, 71]. Although, the exact cause of autoreactive B cell dysregulation is still unknown, the reduction in Treg suppressive capabilities and the concomitant activation of autoreactive B cells may be linked [66, 72]. Finally, there is documented evidence of dysfunctional migratory receptors and cytokines in both MS and MG which are used by Tregs; therefore, repairing migratory defects may be another therapeutic strategy [73, 74].

### Relevant Treg subsets and suppression mechanisms

There are multiple Treg subsets and a variety of suppressive mechanisms which they use. The two main Treg subtypes include natural Tregs (nTreg) and induced Tregs (iTreg), each of which express the FoxP3 transcription factor. The nTreg is generated in the thymus, while iTregs are generated in the periphery, further distinguished by their level of methylation at the FoxP3 promotor [75]. Tregs are considered essential for maintaining peripheral tolerance against self-antigens through a variety of soluble mediators including IL-10, IL-35, and TGF-β, and cell surface molecules such as CD25 and CTLA-4 (rapidly recycled to/from the cell surface) [76–84]. IL-10 has been shown to inhibit T cells by preventing CD28 co-signaling that leads to tyrosine phosphorylation [85]. The predominant suppressive mechanism used by T regulatory type 1 cells (Tr1; CD4^+^IL-10^+^FoxP3^−^), found to be dysfunctional in MS patients, also involves IL-10 [27]. Another Treg subset, namely iTr35, has a suppression mechanism which is not fully understood [86]. However, IL-35 is associated with upregulation of inhibitory molecules programmed cell death protein 1, T cell immunoglobulin and mucin-domain containing-3, and lymphocyte activation protein 3 on T cells [87]. TGF-β has been found to direct antibody production toward non-inflammatory IgG4 and IgA isotypes [85]. Additionally, TGF-β allows for differentiation of iTregs from CD4^+^CD25^−^FoxP3^−^ Teff cells [88]. Notably, iTregs have been induced using other molecules such as retinoic acid [89]. The CTLA-4 and CD28 provide co-stimulatory signals when bound by their cognate ligands CD80 and CD86 expressed on antigen-presenting cells (APCs). T cell co-stimulation with CD28 leads to activation signals; in contrast, co-stimulation with CTLA-4 leads to inhibition signals [90]. CTLA-4 co-stimulation then leads to trans-endocytosis of its ligands into the T cell, thus depleting ligands for use in CD28 co-stimulation [72]. Although CD25 (IL-2Rα) expression is seen on both Tregs and Teff cells, it is constitutively expressed on Tregs while only transiently expressed on Teff cells upon activation [91]. IL-2Rα, when dimerized with the β-chain, gains high affinity for IL-2, thus it is thought to cause IL-2 depletion by Tregs which restricts IL-2-dependent activation of Teff [91]. Lastly, Tregs have been shown to suppress and destroy B cells using perforin which creates pores in the target cell membrane, as well as granzyme B, a serine protease which induces programed cell death via caspase activation [66, 92]. Perforin and granzyme B are important in regulatory T follicular cells (TFR; CD4^+^CXCR5^+^FoxP3^+^) which have been shown to play a key role in suppressing B cells in the GCs and are reduced in MG patients [93, 94].

### Treg dysfunction in MS pathology

MS is a neuroinflammatory disease characterized by demyelination and inflammatory infiltrates in the central nervous system (CNS) [95]. Immunologically, MS correlates with Treg dysfunction, enhanced Th1 and Th17 responses, and autoreactive B cell over activity [95, 96]. This immune disequilibrium may be caused by a loss of Treg suppression of Teff which leads to myelin destruction causing neuronal damage and neuroinflammation [96, 97]. Clinically, MS presents with multiple neurological deficits and is grouped into three categories: 85% of patients are initially diagnosed with a relapsing remitting disease, of which 50–60% progress to secondary progressive MS, having worsening deficits with or without relapse [98]. A small percentage of patients, about 10%, exhibit primary progressive MS, in which symptoms are present from onset and gradually worsen without notable remission [98]. One of the most well-characterized pre-clinical models for MS is the myelin oligodendrocyte glycoprotein (MOG)-induced EAE model [95, 99]. Other common self-antigen-specific EAE models tend to use myelin basic protein or proteolipid protein (PLP); however, MS patients have been suggested to have antibodies against multiple self-antigens, such as heat shock protein α B-crystallin or S100beta [95, 100–103]. MOG-induced EAE is initiated by the injection of either full length recombinant MOG protein, or peptide fragments including a 21 amino acid peptide spanning MOG residues 35-55, together with complete Freund’s adjuvant followed by pertussis toxin injection [95, 99]. After immunization, APCs present MOG peptides to T cells leading to autoreactive T cell activation, migration into the CNS, and subsequent neuroinflammation.

A recent analysis of over 14,000 MS patients using ImmunoChip genotyping found abnormalities in genes involved in Treg IL-2 signaling, CD25, and CD127 [55]. While CD127 is expressed highly on Teff, but minimally on Tregs, molecules such as GITR, OX40, Helios, CD49b are also expressed on Tregs [22, 28, 47, 77]. Furthermore, many MS patients have decreased FoxP3 expression in Tregs, decreased Treg suppressive function, and decreased levels of Tr1 [1, 27]. Additionally, recent research suggests that Tregs are unable to properly infiltrate the CNS during the course of the disease [104–106]. Brain biopsies from MS patients revealed that 30% of the lesions lacked FoxP3 expression [104]. Fas, a cellular apoptotic pathway receptor, is seen upregulated on Tregs in MS brain biopsies suggesting increased susceptibility to apoptosis [104]. Taken together, these findings suggest that Tregs might be restricted from migrating to neuroinflammatory sites or undergo apoptosis upon arrival. Treg dysfunction during the disease progression can also be noted through modulation of CD28 and CTLA-4 co-receptors mentioned earlier. Studies in EAE have shown that during relapse, there is increased expression of CD28, co-localizing with T cell receptor subunit CD3, in the CNS blood vessels and parenchyma [107]. During remission, CD28^+^ cells decrease while CTLA-4^+^ cells increase [107]. Considering Tregs constitutively express CTLA-4 while Teff do not, it is possible these cells are Tregs; however, the study did not look for co-expression of FoxP3 [107, 108]. Taken together, Treg abnormalities in MS appear to involve loss of suppressive capacity as well as defect in migration into the CNS.

Many treatments which increase Tregs in EAE have been found to be efficacious, while other treatments, although successful, showed no effect on Tregs [109]. Administration of an indoleamine 2,3-dioxygenase (IDO) metabolite which increases Treg number leads to significant amelioration of MOG-induced EAE [110]. Additionally, the effect of IL-10-based therapy has been evaluated in different EAE models; most resulted in reduced clinical scores, perhaps associated with an increase in Tr1 associated with IL-10 administration [111, 112]. Some of the most affirmative evidence advocating Treg augmenting treatments have utilized adoptive transfer techniques. Adoptive transfer of FoxP3^−^ Treg cells that ectopically expressed the transcription factor FoxA1 (suggested marker for a novel Treg) ameliorated EAE in IFN-β knockout (KO) mice and these cells were increased in response to treatment of MS patients with IFN-β, suggesting a role for this Treg subset [113]. Although adoptive transfer of CD4^+^CD25^+^FoxP3^+^ cells reduced EAE, they differed in their efficacy. Adoptive transfer of CNS-derived KLRG1^−^ and KLRG1^+^ cells modestly reduced the clinical score at the termination of the experiment without affecting peak response (KLRG1 is associated with natural killer cells but was tested due to its increased expression on Tregs when entering the CNS of EAE mice) [114]. Likewise, CNS-derived CD4^+^CD25^+^ cells reduced the peak clinical EAE score [115]. Finally, experiments knocking out known Treg proliferating molecules, such as IDO or IL-10, resulted in exacerbation of EAE [110, 116].

In contrast to the above findings, *FoxP3gfp* knock-in mice had an accumulation of Treg cells in the CNS, but it failed to control EAE [117]. Administration of cholesterol-reducing statins such as atorvastatin inhibited the development of EAE without increasing the frequency of FoxP3^+^ Treg cells or Th2 cells, yet there was a substantial increase in IL-10 expression [109]. Taken together, these data tend to suggest that modulation of FoxP3 Tregs may not always be necessary for the protective effect of certain treatment modalities for EAE. Inasmuch as administration of statins involve non-specific immunosuppression, it is hard to avoid undesirable consequences. Therefore, it appears that boosting the Treg cells may produce a desirable clinical outcome in MS.

### Treg migratory receptor dysregulation may have implications in MS

Dysfunctional migration of Tregs to neuroinflammatory sites could have profound implications; for example, GCN2 (aids in CCL2-mediated migration) KO Tregs adoptively transferred into EAE mice were unable to enter the CNS and hence mice were unable to undergo remission [118]. Some chemoattractants (e.g., CCL17) or their receptors (e.g., CCR4 and CXCR3) appear to facilitate Treg homing to neuroinflammatory sites. Human CD4^+^CD25^+^ Treg cells express CCR4 which respond to mature dendritic cell-derived CCL17 and are thereby recruited to the site of inflammation. Butti et al. determined that CCR4 is indispensable for Treg recruitment into the cerebral spinal fluid (CSF) and that recruitment was necessary for EAE amelioration likely due to the increased CCL17 levels after administration of IL-4 gene therapy into the CSF [119]. Studies of malignant diseases also find a correlation between the levels of CCL17 and accumulation of FoxP3+ cells, implicating CCL17 as a Treg recruiter molecule [120, 121]. Therefore, CCL17 administration at specific sites may be utilized to recruit Tregs.

Molecules such as CXCR3 are important for Tr1 and Treg migration to the CNS [106, 122]. The chemokine receptor CXCR3 that promotes trafficking of activated T and natural killer cells in response to CXCL9, CXCL10, and CXCL11 is expressed heavily on Tr1 cells which can direct Tr1 to the CNS thereby implying a role in EAE [122]. Administration of CXCL10-Fc exacerbates EAE, whereas CXCL11-Fc leads to amelioration with subsequent polarization to Tr1 [122]. Since CXCR3 and CXCL10 are found elevated in neuroinflammatory sites in MS patients, over-recruitment of Teff may be occurring, thus use of CXCL11 could be a strategy to recruit additional Tr1 to these neuroinflammatory sites [123]. Additionally, CXCR3 KO mice have more severe EAE with reduced FoxP3^+^ cells in the CNS despite little change in total CD4^+^ infiltrates implying that CXCR3 may be crucial for Treg migration to the CNS [106]. Therefore, a strategy to increase CXCR3^+^ expression on FoxP3^+^ Tregs may be useful for recruitment to the CNS. Enhanced CXCR3 expression by Tregs has been seen with the use of IL-27, thus providing a treatment strategy to facilitate selective Treg migration [122, 124].

### Therapeutics for multiple sclerosis which restore Treg abnormalities

Many common MS treatments could result in adverse effects revealing a need for new therapeutic strategies [9, 125]. Additionally, current treatments such as IFN-β (Avonex, Rebif, and Betaseron), glatiramer acetate (GA; Copaxone), fingolimod (Gilenya), teriflunomide (Aubagio), dimethyl fumarate (Tecfidera), Natalizumab (Tysabri), and mitoxantrone, although beneficial, are not curative [9]. Corticosteroids, which have been in use for over 75 years, are still the treatment of choice for acute MS symptoms [9, 126]. However, they lack specificity and tend to cause severe adverse effects such as abnormal weight gain, behavioral changes, oral thrush, and others [126]. Over two decades ago, glatiramer acetate became one of the first treatments for reducing relapse rates in MS patients; however, it had only a modest efficacy in reducing disease activity [127]. Many newer treatments, such as the ones indicated above, have been implemented since then, yet nearly all can cause significant side effects [9, 128, 129]. Therefore, there is a need for novel treatments which reduce disadvantages of current treatments while providing greater efficacy.

**Fig. 1:**
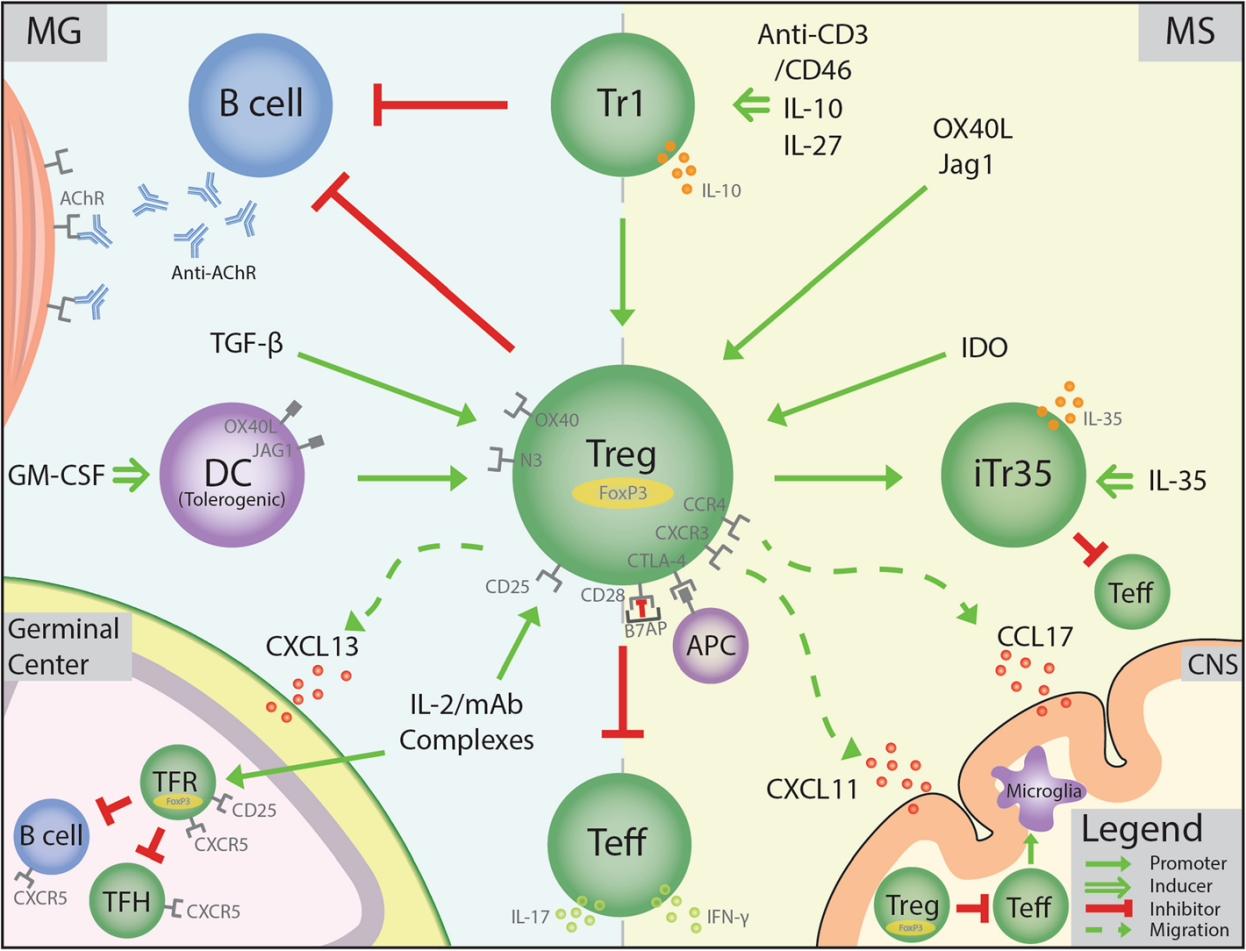
MG and MS treatment schemes aimed at augmenting Tregs based on experimental models. AChR: acetylcholine receptor; APC: antigen-presenting cell; B7AP: B7 antisense peptide; Bimolecular peptide inhibitor; CNS: central nervous system; DC: dendritic cell; GM-CSF: granulocyte macrophage-colony stimulating factor; IDO: indoleamine 2,3-dioxygenase; iTr35: induced T-regulatory 35 cell; IFN-γ: interferon gamma; MG: myasthenia gravis; MS: multiple sclerosis; N3: Notch 3 receptor; IL: interleukin; Teff: effector T cell; TFH: follicular helper T cell; TFR: regulatory T follicular cell; TGF-β: transforming growth factor beta: Tr1: T-regulatory 1 cell; Treg: T-regulatory cell

Most of the clinical treatments do not target Tregs as specifically as new pre-clinical treatments can [130]. Despite different targets, some treatments have been shown to increase Treg numbers such as IFN-β, GA, fingolimod, and teriflunomide, while others, such as dimethyl fumarate, natalizumab, mitoxantrone, and corticosteroids, have resulted in reduced, unaffected, or contradictive effects on Treg numbers [131–140]. Treatments such as the lipid-lowering drug atorvastatin mentioned earlier have no effect on the FoxP3 Treg population, indicating that increasing the Treg population, although may be desirable, may not always be necessary [109]. However, this review will discuss strategies to restore Treg abnormalities as therapeutic modalities that may be considered for further testing. These Treg abnormalities have been corrected using cytokines such as IL-10, IL-27, IL-35, bimolecular protein inhibitors (BPIs), indoleamine 2,3-dioxygenase (IDO), or the chemokine CXCL11 (Table 2; Fig. 1) [80, 106, 110, 111, 141]. Additionally, we propose modalities of treatment for further testing. These include Jagged-1/OX40L co-treatment and site-specific CCL17 administration for Treg recruitment (Table 2).

**Table 2:** Multiple sclerosis treatment approaches using Treg augmentation based on pre-clinical models

Therapeutic modality	Intended Treg augmentation	Approach	Outcome	Reference
IL-10	Upregulate Tr1, increase Tregs through DC modulation	EAE (MOG 1-125) Dark Agouti rats. pcDNA IL-10 Gene therapy on day 0 and 3	↓ clinical score ↓ sensory loss ↓ microglial/ macrophage and astrocyte activation	[143]
IL-35	Induction of iTr35	EAE (MOG 35-55) C57BL/6. Adoptive transfer of iTreg induced with rIL-35	↓ clinical score ↑ life span ↑ iTr35	[80]
Bifunctional Peptide Inhibitors	Inhibit CD28 co-stimulation to promote CTLA-4 co-stimulation	EAE (MOG 35-55) C57BL/6. Injection of B7AP-PLP (anti-CD28 linked to PLP; 100 nmol) on days 4, 7, and 10	No clinical signs ↓ change in weight ↓ IL-6,-17 ↑ IL-2, -4, -5	[155]
IDO Metabolite	Increase Tregs, increased CCL2-mediated migration to CNS	EAE (MOG 35-55) C57BL/6. 3-HAA (downstream IDO metabolite) treatment daily	↓ clincal Signs ↓ disease peak ↑ FoxP3+ Tregs ↓ IL-17, IFN-gamma	[110]
CXCL11	Increase Tr1 migration to CNS, increase IL-10 expression, polarize Tr1	EAE (MOG 35-55 and PLP129-151) in C57BL/6 and SJL/j mice, respectively. CXCL11-IgG every other day and adoptive transfer of CD4+ cells from CXCL11-IgG treated EAE SJL/j mice	↓ clinical score ↓ histological score Prevented relapse ↑ Tr1 ↓ IFN-gamma, IL-17	[122]
IL-27	Proliferation of Tr1, upregulate CXCR3 on FoxP3 Tregs for migration to CNS	EAE (MOG 35-55) C57BL/6. Adoptive transfer of CD4+ cells treated with MOG, IL-12, and IL-27 (control: MOG and IL-12)	↓ clinical score ↑ Tr1	[154]
		WT C57BL/6 J. Injection of IL-27 DNA plasmids	↑ CXCR3 on Tregs and not Teff	[124]
Emperically supported treatments using EAE and other experimental data				
OX40L Jagged-1 Co-treatment	Selectively expand Tregs in TCR-independent manner, activate CD46 for induction of Tr1	EAE (MOG 35-55) C57BL/6. Jagged1-Fc on days 0, 2, 4, 6, 8	↓ clinical scores ↓ disease peak ↑ IL-10, -4	[159]
		EAE (PLP 139-151) SJL/j. Alpha OX40 agonist on days 10, 12, and 14.	↓ clinical scores ↑ Tregs ↑ IL-2, -6, -17, and IFN-g in the CNS	[167]
Site-specific CCL17 Injection	Selectively recruit Tregs to neuroinflammatory sites via CCR4	EAE (MOG 35-55) C57BL/6. IL-4 gene therapy injection into cisterna magna on day of onset (12-16 days)	↓ clinical scores ↑ IL-4 ↑ CCL17 ↑ Tregs in brain and spinal cord	[119]
		Ex vivo human Treg transmigration assay with porcine aortic endothelial cells coated with CCL17	↑ Treg adhesion ↑ Treg transmigration via CCR4	[172]

*CCL* chemokine ligand, *CCR* chemokine receptor, *CTLA-4* cytotoxic T-lymphocyte-associated protein 4, *DC* dendritic cell, *EAE* experimental autoimmune encephalomyelitis, *IFN* interferon, *IL* interleukin, *iTr35* induced T-regulatory 35 cell, *MOG* myelin oligodendrocyte glycoprotein, *MS* multiple sclerosis, *PLP* proteolipid protein, *Tr1* T-regulatory 1 cell, *Treg* T-regulatory cell

#### Administration of interleukins

IL-10 mediated suppression is regarded as a main mechanism of Treg suppression. IL-10 therapies have resulted in a reduction of symptoms in majority of the EAE models tested (reviewed in reference [111]) [142, 143]. Despite the success in pre-clinical models, it has not been as successful in clinical trials conducted in other autoimmune diseases such as Crohn’s disease and rheumatoid arthritis [144, 145]. Focus might instead be diverted toward IL-10 replenishing strategies which down modulates the Fas-mediated apoptosis both in Tregs and Bregs [111, 145]. Research shows increasing IL-10 expression may be done through upregulating IL-10 producing Tr1, increasing Treg number, or through induction of tolerogenic DCs (CD11c^+^CD8α^−^) [27, 67, 110]. Since many documented positive correlations between IL-10 and MS remission have been documented, and IL-10 secreting Tr1 usage in pre-clinical models appears useful, upregulation of Tr1 cells could be a useful IL-10 replenishment strategy which would also rectify Tr1 defects in MS [146, 147].

IL-27 and IL-35 are anti-inflammatory cytokines that can regulate Treg responses [148, 149]. IL-35 has been shown to play a key role in FoxP3+ Treg suppression and in the induction of iTr35 [80, 149]. Treatment with IL-35 has shown to lead to EAE amelioration with concomitant induction of iTr35 (Fig. 1) [80]. These iTr35 have also been shown to ameliorate other autoimmune models such as collagen-induced arthritis and IBD [150]. IL-35 is not produced by human FoxP3 Tregs, but ex vivo experiments on human T cells have been able to induce iTr35 suggesting that it may have a similar role in humans [150, 151]. IL-27 has both inflammatory and anti-inflammatory pathways. IL-27 activates Th1 response through T-bet signaling; however, it also inhibits Th17 development and induces IL-10 production [152]. IL-27 role in FoxP3 Tregs is less understood, yet it has been shown to induce Tr1 production in vivo leading to EAE amelioration [153]. Administration of exogenous IL-27 to EAE mice has also been shown to reduce IL-17 production, Th17 cells, and CNS infiltration [153, 154]. Similarly, IL-27R-deficient mice are prone to develop more severe EAE [153]. Thus, IL-27 and IL-35 act to induce other Treg subsets, one of which, Tr1, is downregulated in MS patients [27].

#### Bifunctional peptide inhibitors

Bifunctional peptide inhibitors (BPIs) have been efficacious in the treatment of EAE [155]. BPIs consist of a self-antigen (i.e., PLP) bonded to an antagonistic peptide usually resembling CD28, LFA-1, or ICAM-1 which are connected by a peptide linker [155]. B7AP was the first BPI tested in EAE; it works by antagonistically binding to B7 (CD80 or CD86) on B cells preventing CD28 binding by Tregs or other T cells (Fig. 1) [155]. Instead, BPIs force CTLA-4-B7 interaction leading to tolerogenic signaling resulting in immune tolerance likely due to the constitutive expression of CTLA-4 on Tregs [78, 155, 156]. This method was originally non-antigen-specific, but recently it was used in combination with PLP which attaches to MHC class-II on B cells leading to an antigen-specific tolerogenic response [155]. Since B7AP itself repressed EAE, although to a lesser extent, the specificity of this approach remains enigmatic [155]. Nevertheless, it was observed that when paired with the self-peptide PLP, the BPI prevented PLP-EAE [155]. Additionally, immune suppression was accompanied by reduced IL-17 and transient suppression of IL-6, while increasing IL-2 and IL-4 expression [155]. It remains to be evaluated whether BPIs can be utilized to induce tolerance against myelin antigens.

#### OX40L/Jagged-1 co-treatment

OX40L and Jagged-1 co-treatment may be beneficial for EAE (Fig. 1). We have shown that OX40L and Jagged-1 expression on GM-CSF-induced bone marrow-derived dendritic cells can increase Tregs and ameliorate experimental autoimmune thyroiditis [157]. Our recently published data also reveals that co-treatment with soluble ligands OX40L and Jagged-1 can successfully delay the onset of diabetes in non-obese diabetic mice [158]. Jagged-1 expression on astrocytes was shown to upregulate TGF-β while decreasing IFN-γ, TNF-α, and IL-17 expression, thus implying a role for Jagged-1 in CNS homeostasis [159]. Stidworthy et al. have shown that Jagged-1/Notch-1 pathway is correlated with re-myelination of neurons, possibly providing a disease-specific advantage for Jagged-1 treatment in EAE [160]. While blocking Jagged-1 has led to EAE exacerbation and reduced Tr1, administration of Jagged-1-Fc has led to EAE amelioration and a subsequent increase in Tr1 [159]. Recently, CD46 was shown to be a receptor for Jagged-1 and CD46 activation induced a switch from inflammatory IFN-γ release to IL-10 expression by Tregs [161]. The role of CD46 in MS has been well documented, additionally, an experiment with cyanomolgus monkeys having MS-like disease revealed a tenfold reduction in CD46-mediated IL-10 expression [162, 163]. CD46 also enhances CD25 expression and elevates granzyme B production [164]. Furthermore, since activation via co-stimulation of CD3/CD46 has led to Tr1 generation, it may be possible to do so using Jagged-1 to activate CD46 [165]. Jagged-1 treatment is successful in other pre-clinical models of autoimmune disease; however, our recently published data revealed this effect on Tregs appears more potent when given along with OX40L [157, 158, 166]. The effect of OX40L administration in EAE appears to depend on the cytokine milieu. One report found administration prior to disease onset suppressed EAE, while giving OX40L early after onset exacerbated EAE [167]. Thus, OX40L and Jagged-1 co-treatment may both increase Treg numbers and rectify Tr1 deficits in MS patients.

#### Migratory modulation

CXCR3 is a migratory receptor expressed on many cells including Tr1 cells (Fig. 1). CXCR3 has been found to be upregulated in demyelinating MS lesions [123]. As mentioned earlier, CXCL11, a CXCR3 chemokine, attracted Tr1 to CNS neuroinflammatory sites upon administration and suppressed EAE while reducing IL-17 and IFN-γ without notable relapse [106, 122]. In addition to recruitment of Tr1, IL-27 administration has caused a modest increase of CXCR3 expression on FoxP3^+^ Tregs, specifically, and could be a strategy to recruit FoxP3^+^ Tregs into the CNS [124].

IDO is reported to cause Treg cell differentiation and Tregs positively influence its expression [168]. IDO is expressed by DCs, and interaction with CTLA-4 has caused upregulation of IDO release [168]. IDO release leads to tryptophan depletion which in turn induces DCs through the GCN2 pathway [169]. GCN2-deficient mice are shown to have reduced Tregs infiltrating the CNS due to impaired detection of the CCL2 gradient [118]. Furthermore, knocking out or inhibiting IDO has led to reduced Treg numbers and EAE exacerbation, suggesting a role for IDO in Treg homeostasis [110, 170]. Thus IDO administration might promote the GCN2 pathway such that it causes Treg migration to the CNS.

As discussed earlier, chemotactic factor CCL17 used by Tregs might be successfully used as a site-specific recruiter for Tregs directly to the site of neuroinflammation (Fig. 1) [119, 171]. Its receptor CCR4 has been shown by Butti et al. to selectively recruit Tregs with great suppressive functionality to the CSF, ameliorating EAE [119]. Studies in other diseases have found a direct correlation with the level of CCL17 and accumulation of FoxP3 [120, 121]. Lastly, porcine endothelial cells secreting CCL17 resulted in enhanced recruitment and transmigration of human Tregs ex vivo [172]. Taken together, recruitment of Tregs to the CNS using site-specific CCL17 administration provides another strategy for Treg recruitment (Fig. 1; Table 2).

### Treg dysfunction coincides with autoantibody production in myasthenia gravis

MG is a NMJ disorder characterized by worsening muscle weakness with sustained contraction which improves upon rest [2]. Damage to the NMJ ion channels is autoantibody mediated where autoantibodies bind and result in the formation of the membrane attack complex consisting of C5b, C6, C7, C8, and C9 leading to muscular membrane damage and muscle weakness [2, 173]. The most common autoantibodies involved are anti-AChR antibodies presenting in 85% of patients, anti-muscle-specific kinase presenting in 5–8%, and anti-low density lipoprotein receptor-related protein in 2–46% of patients [69, 174, 175]. Anti-AChR autoantibodies bind to the AChR at the NMJ which induces complement-mediated damage of the NMJ, cause production of inflammatory cytokines, and subsequent reduced muscle functionality. The autoreactive T cells, found both in MG and EAMG, provide the necessary help for the pathogenic autoantibody production [2]. Interestingly, 60% of MG patients develop thymic hyperplasia and 10% develop thymomas, suggesting T cell dysregulation as an etiology [176]. EAMG, the experimental model for MG, is typically induced upon immunization with *Torpedo californica* AChR emulsified in an adjuvant [177]. The EAMG model is reported to closely resemble the clinical, pharmacological, histological, electrophysical, immunological, and pathogenic mechanisms in MG, yet it does not show thymic hyperplasia characteristic of human MG [2, 178, 179]. Since its description in the early 1970s, the EAMG model has been indispensable in understanding MG [180].

Many studies have reported Treg dysfunction in MG [13, 28]. Data is conflicting whether reduced Treg numbers contribute to the disease pathogenesis [60, 181]. A recent genome-wide analysis of MG patients revealed CTLA-4 and RANK (receptor activator of nuclear factor-kappa B) gene loci to be associated with disease risk [56]. CTLA-4, implicated in Treg-mediated suppression as discussed earlier, is found downregulated in MG patients [182]. RANK ligand (RANKL), which is preferentially expressed on Tregs as compared to Teff [183], is thought to function by suppressing CD11c^+^ DC activation through the RANK-RANKL pathway [184]. Inhibition of RANKL hinders Treg expansion, whereas RANKL upregulation has been seen to induce Treg proliferation [184, 185]. Further, Tregs in MG patients have decreased expression of FoxP3 and IL-10 indicating a functional deficit, while they also have enhanced expression of Fas indicating increased susceptibility to Fas-mediated apoptosis [13, 28]. Immunologically, reduced Treg suppressive activity in MG patients is accompanied by elevated inflammatory cytokines (IL-6, IL-17, TNF-α, IL-12, and IL-1β), most of which are normally suppressed by Tregs [2, 28]. Decreased suppression from Tregs may also correlate with autoantibody production. We and others have shown that Tregs adoptively transferred into EAMG mice have significantly delayed disease progression, reduced numbers of autoreactive T cells, and lower levels of AChR antibodies [59, 67]. Adoptive transfer of Tregs from GM-CSF-treated EAMG mice also resulted in a reduction of pro-inflammatory cytokines IL-6, IL-17, and IFN-γ, and increase in FoxP3 and IL-10 expression [67]. Reduction in clinical scores and AChR antibodies after Treg adoptive transfer was likely due to reduced complement fixation, secondary to suppression of B cells by Tregs as was seen in a model of systemic lupus erythematosus (SLE) [92]. A possible mechanism by which Tregs might reduce autoantibody production and subsequent complement fixation is via destruction of autoreactive B cells by contact-dependent release of perforin and granzymes [66]. Taken together, addressing Treg abnormalities in MG patients may provide a strategy for therapeutic intervention.

### What recent research reveals about Treg homing to germinal centers in myasthenia gravis

Few studies have explored Treg migratory patterns in EAMG or MG, yet one chemokine receptor, CXCR5, involved in Treg migration has been implicated in MG [74, 186]. CXCR5 is used by follicular T helper cells (TFH; CD4^+^CXCR5^+^FoxP3^−^), TFR, and B cells to facilitate homing to the B cell zone in GCs [187]. The TFR can suppress TFH and B cell numbers in the GC; however, MG patients have a documented reduction in TFR cells along with a reduced TFR to TFH cell ratio [93, 188]. Furthermore, the ratio of TFR to TFH cells is found to be inversely correlated with clinical severity in patients treated with corticosteroids [93]. Thus, restoring the TFR to TFH imbalance would be useful for Treg-based therapeutics, and this could be achieved with the use of IL-2/mAb as seen in an experiment using EAMG [93, 189].

### Anti-inflammatory therapeutics for myasthenia gravis which augment Treg function

MG has a limited range of treatments; the clinical treatments usually include acetylcholinesterase inhibitors (pyridostigmine bromide), rituximab (anti-CD20), corticosteroids, immunosuppression (such as azathioprine), intravenous immunoglobulin (IVIG), plasmapheresis, and thymectomy, yet none focus on curing the underlying cause [11]. Most current treatments are unsuccessful in stopping disease reoccurrence after remission and may have unacceptable side effects after long-term use [11]. Although the mechanism is not understood, Tregs have been seen to modestly increase with the use of drugs not targeting Tregs, such as pyridostigmine, rituximab, azathioprine, and IVIG [190–193]. Corticosteroids lack specificity and importantly cause severe adverse effects such as bruising, abnormal weight gain, behavior changes, oral thrush, and others [194]. Pyridostigmine bromide, the most commonly used acetylcholinesterase inhibitor, is the first line for symptomatic treatment [195]. Yet it provides only short-term relief, it is not a disease-modifying therapeutic (DMT), and it can cause a hypersensitivity rash [195]. Azathioprine, a commonly used DMT, causes side effects in roughly 20% of patients and failed to prevent relapse in roughly 33% of patients in a long-term study of 117 MG patients [196]. Thymectomy has proven to be a beneficial DMT; however, it does not cure the underlying disease, and patients still suffer side effects from other treatments because thymectomized patients still require additional interventions such as corticosteroids or azathioprine [197]. Taken together, there is a need for more efficacious DMTs focused on destroying autoreactive B cells to reduce autoantibody production and NMJ destruction. Although anti-B cell therapies are in practice like rituximab (anti-CD20 antibody), Tregs’ ability to destroy autoreactive B cells may provide relatively better specificity [68, 92]. Treg-augmenting therapies which might accomplish this task or treat MG Treg abnormalities include GM-CSF, IL-2/mAb complexes, OX40L-Jagged-1 co-treatment, and TGF-β administration.

#### GM-CSF

GM-CSF treatment of MG has led to a reduction of clinical signs and symptoms while increasing Tregs [198]. In EAMG, we have determined there are numerous benefits from GM-CSF treatment, namely, increasing the number of Tregs, halting antigen-specific T cell proliferation, enhancing IL-10 production, suppressing B cell proliferation, reducing anti-AChR antibody production, and reducing expression of IL-6, IL-17, and IFN-γ [67, 199]. We have showed that GM-CSF can induce tolerogenic semi-mature DCs (CD11c^+^CD8a^−^) which cause expansion of Treg cells that suppress EAMG [200]. Such GM-CSF induced Tregs likely suppressed autoimmunity through the secretion of IL-10, as we have shown in an animal model of thyroiditis [201]. Ex vivo co-culture of GM-CSF exposed bone marrow DCs with CD4^+^ T cells induced selective expansion of Tregs via OX40L and Jagged-1-mediated signaling [200, 202]. Alternatively, it has also been shown that GM-CSF can directly bind to its receptor expressed on human Tregs leading to their expansion [203]. In all, GM-CSF treatment may rectify multiple Treg abnormalities (Table 3).

**Table 3:** Myasthenia gravis treatment approaches using Treg augmentation based on pre-clinical models

Therapeutic modality	Intended Treg augmentation	Approach	Outcome	Reference
GM-CSF	Expand functional Tregs via Tolerogenic DCs	77-year-old male with myasthenia crisis untreated with conventional treatments. GM-CSF 750 μg daily for 2 days, then 250 μg daily for 3 days, then 5 more 250 μg doses daily in week 7–8	Cessation of myasthenic crisis ↑ strength weaned from ventilator	[198]
		EAMG (tAChR) in C57BL/6. GM-CSF daily on days 0–9 and 37–41	↓ clinical score ↓ weight loss ↓ anti-AChR Ab ↑ IL-10, -4, FoxP3 ↓ IFN-g	[200]
IL-2/mAb complexes	Activate peripheral Treg, activate and TFR in GC to suppress TFH and B cells, increase Treg migration to GC	EAMG (tAChR) in thymectomized C57BL/6. IL-2 complexes twice weekly	↓ clinical score ↓ autoantibodies ↓ CD19 cells ↑ TGF beta ↓ IFN-g	[189]
Emperically supported treatments yet to be used in EAMG pre-clinical models				
OX40L Jagged-1 Co-treatment	Selectively expand functional Tregs in TCR-independent manner, modulate CD46 Treg stimulation	EAMG (tAChR) C57BL/6. Adoptive transfer of Tregs from GM-CSF treated EAMG mice into EAMG mice	↓ clinical score ↓ auto antibodies ↑ AChR content	[67]
		Experimental autoimmune thyroiditis (via murine thyroglobulin) CBA/j. Adoptive transfer of OX40L + Jagged1+ from GM-CSF treated bone marrow DCs (control: non treated and single OX40L positive)	↓ pathology in double positive only ↑ Treg in double positive only	[157]
TGF-beta	Induce iTregs	Lupus-prone mice (NZB/NZW F1). Adoptive transfer of CD4 + CD62L + CD25-CD44^low^ cells stimulated with anti-CD3 and anti-CD28 in presence of IL-2 and TGF-beta	↓ multiple auto antibodies	[208]
		Ex vivo human MG peripheral blood mononuclear cells. Stimulated with TGF-beta	↓ mRNA for IFN-gamma, IL-4, -6, TNF alpha, TNF beta Suppressed AChR-reactive IFN-gamma and IL-4 secreting cells	[209]

*AChR* acetylcholine receptor, *DC* dendritic cell, *EAMG* experimental autoimmune myasthenia gravis, *GC* germinal center, *GM-CSF* granulocyte macrophage-colony stimulating factor, *IFN* interferon, *mAb* monoclonal antibody, *MG* myasthenia gravis, *TFH* helper follicular T cell, *TFR* regulatory T follicular cell, *TGF* transforming growth factor, *TNF* tumor necrosis factor, *Treg* T-regulatory cell

#### Inhibit B and TFH cells at GCs

The thymus plays a critical role in anti-AChR antibody production and MG pathogenesis [204]. B cells are implicated not only because of autoantibodies but also because of increases in CXCL13, CCL21, and BAFF in the thymus of MG patients, all of which lead to B cell activation [205]. CXCL13 is utilized by B cells, TFH, and TRH cells to migrate to GCs using CXCR5. Thus, a therapeutic opportunity would be to increase Treg migration to the GC to suppress activated TFH and B cells. This may be achieved with IL-2/mAb complexes (monoclonal antibody greatly increases IL-2 half-life) that have ameliorated EAMG while reducing CD19^+^ cells, autoantibody levels, and IFN-γ expression [189]. The effects of IL-2/mAb complexes on Tregs and migration to GCs are better understood through an experiment using chronic graft-versus-host disease (cGVHD) in which administration of IL-2/mAb complexes increased splenic Tregs and TFR while ameliorating cGVHD without increasing TFH [94]. Adoptive transfer of wild type Tregs without IL-2/mAb administration caused a reduction in B cell number in the GC dependent upon CXCR5^+^ Tregs homing to GCs [94]. These findings in GVHD may be relevant to MG as GCs as well as B and T cells are also implicated in GVHD pathogenesis [206]. A mechanism by which IL-2/mAb may act on Tregs over Teff is through the use of IL-2Rα, CD25. The high affinity Tregs have for IL-2 through their CD25 expression will deplete IL-2 from the surrounding milieu restricting IL-2-dependent Teff activation [91]. It should be noted that IL-2/mAb complexes, when used in mice with acute GVHD, resulted in rapid death of mice within 4 days with concomitant increases in Teff, thus the efficacy of treatment may depend on the level of immune activation [94]. Therefore, using IL-2/mAb complexes may be an effective strategy to activate and proliferate Tregs and/or TFR causing suppression and reduction of TFH and B cells in the GC (Fig. 1; Table 3).

#### OX40L/Jagged-1 Co-treatment

As stated earlier, GM-CSF treatment induced Treg expansion via OX40L and Jagged-1 release from tolerogenic DCs (Fig. 1) [157, 200]. Considering OX40 is found to be upregulated in MG, and our recently published data shows that soluble OX40L and Jagged-1 co-treatment can delay autoimmune diabetes in NOD mice while increasing Tregs, EAMG could be a successful candidate for OX40L and Jagged-1 co-treatment [157, 158, 207]. More interestingly, CD46, a complement receptor expressed on many cell types, is found to co-stimulate with Jagged-1 leading to Treg activation as well as induction of Tr1 cells [161]. Although the role of Tr1 cells is unknown in MG, their suppressive capabilities have been well documented in other autoimmune models [147]. Thus, CD46 activation via Jagged-1 may provide some novel insights into MG treatment modalities [39]. In conclusion, OX40L and Jagged-1 treatment may lead to an increase in Tregs, or even Tr1 cells and rectify OX40 or CD46 dysregulation in MG (Table 3).

#### TGF-β

TGF-β is a potent activator of Tregs leading to iTreg generation [208]. Generation of iTregs ex vivo in the presence of TGF-β when cultured with B cells from lupus-prone mice (NZB/W) has been found to induce apoptosis of B cells, reduce B cell activation, and reduce autoantibody levels [208]. Additionally, adoptive transfer of iTregs resulted in a greater reduction of autoantibodies compared to nTregs (not exposed to TGF-β ex vivo) [208]. Ex vivo, the addition of TGF-β to mononuclear cells from MG patients induced suppression of cells autoreactive to AChR [209]. The blocking of complement receptors C3a and C5a has been shown to induce expression of TGF-β and IL-10 leading to generation of iTregs which when adoptively transferred potently suppressed the disease [210]. Other studies have also suggested a link between reduced complement expression with increased Treg numbers [211, 212]. Additionally, TGF-β has been shown to reduce iNOS production, again leading to concomitant Treg proliferation [213]. Taken together, it appears TGF-β could restore immunological imbalance in MG patients.

## Conclusions

Current MS and MG treatments have modest long-term efficacy and are often associated with severe side effects that fail to treat the underlying disease. Although current treatments do not tend to act on Tregs, they provide a method for regulating autoimmune activation. The Treg augmentation therapies discussed herein focus on rectifying Treg abnormalities by enhancing Treg suppressive activity and/or numbers, increasing Treg migration, causing Treg-dependent B cell destruction or suppression, or enforcing tolerogenic signals from Tregs. Therapies which increase Treg suppressive activity and migration may be particularly useful for MS, whereas MG Treg augmenting therapies should focus on controlling autoreactive B cells. Finally, because these Treg therapeutics appear to act via different mechanisms (Fig. 1), it might be possible to provide synergistic benefits if combined with other tested pre-clinical treatments in MS or MG.

## Abbreviations

AChR: Acetylcholine receptor
APC: Antigen presenting cell
B7AP: B7 antisense peptide
BPI: Bifunctional peptide inhibitor
CCL: Chemokine ligand
CCR: Chemokine receptor
CNS: Central nervous system
CSF: Central spinal fluid
CTLA-4: Cytotoxic T-lymphocyte-associated protein 4
DC: Dendritic cell
DMT: Disease-modifying treatment
EAE: Experimental autoimmune encephalomyelitis
EAMG: Experimental autoimmune myasthenia gravis
FoxP3: Forkhead box P3
GBS: Guillain-Barre syndrome
GC: Germinal center
GCN2: General control nonderepressible 2
GM-CSF: Granulocyte macrophage-colony stimulating factor
GVHD: Graft versus host disease
IDO: Indoleamine 2,3-dioxygenase
IFN: Interferon
IL: Interleukin
iTr35: Induced IL-35 T-regulatory cell
iTreg: Induced T-regulatory cell
MG: Myasthenia gravis
MOG: Myelin oligodendrocyte glycoprotein
MS: Multiple sclerosis
NMJ: Neuromuscular junction
nTreg: Natural T-regulatory cell
PLP: Proteolipid protein
RANK: Receptor activator of nuclear factor-kappa B
SLE: Systemic lupus erythematosus
Teff: T-effector cell
TFH: Helper follicular T cells
TFR: Regulatory follicular T cells
TGF-β: Transforming growth factor beta
Th1: T-helper 1 cell
Th17: T-helper 17 cell
TNF: Tumor necrosis factor
Tr1: T-regulatory 1 cell
Treg: T-regulatory cell
